# Glucocorticoid excess and COVID-19 disease

**DOI:** 10.1007/s11154-020-09598-x

**Published:** 2020-10-06

**Authors:** Valentina Guarnotta, Rosario Ferrigno, Marianna Martino, Mattia Barbot, Andrea M. Isidori, Carla Scaroni, Angelo Ferrante, Giorgio Arnaldi, Rosario Pivonello, Carla Giordano

**Affiliations:** 1 Dipartimento di Promozione della Salute, Materno-Infantile, di Medicina Interna e Specialistica di Eccellenza “G. D’Alessandro”, UOC di Malattie endocrine, del Ricambio e della Nutrizione, Università degli studi di Palermo, Piazza delle Cliniche 2, 90127 Palermo, Italy; 2 Dipartimento di Medicina Clinica e Chirurgia, Sezione di Endocrinologia, Università Federico II di Napoli, Via Sergio Pansini 5, 80131 Naples, Italy; 3 Clinica di Endocrinologia e Malattie del Metabolismo, Dipartimento di Scienze Cliniche e Molecolari (DISCLIMO), Università Politecnica delle Marche, Ospedali Riuniti di Ancona, Via Conca 71, 60126 Ancona, Italy; 4 Endocrinology Unit, Department of Medicine, DIME University-Hospital of Padova, Padua, Italy; 5 Department of Experimental Medicine, Policlinico Umberto I, COVID Hospital, Sapienza University of Rome, 00161 Rome, Italy; 6 Dipartimento di Promozione della Salute, Materno-Infantile, di Medicina Interna e Specialistica di Eccellenza “G. D’Alessandro”, UO di Reumatologia, Università degli studi di Palermo, Palermo, Italy

**Keywords:** Cushing’s syndrome, SarsCoV2, Glucocorticoid, Infections, Cortisol, Immune system

## Abstract

The pandemic of coronavirus disease (COVID-19), a disease caused by severe acute respiratory syndrome coronavirus 2 (SARS-CoV-2), is causing high and rapid morbidity and mortality. Immune system response plays a crucial role in controlling and resolving the viral infection. Exogenous or endogenous glucocorticoid excess is characterized by increased susceptibility to infections, due to impairment of the innate and adaptive immune system. In addition, diabetes, hypertension, obesity and thromboembolism are conditions overrepresented in patients with hypercortisolism. Thus patients with chronic glucocorticoid (GC) excess may be at high risk of developing COVID-19 infection with a severe clinical course. Care and control of all comorbidities should be one of the primary goals in patients with hypercortisolism requiring immediate and aggressive treatment. The European Society of Endocrinology (ESE), has recently commissioned an urgent clinical guidance document on management of Cushing’s syndrome in a COVID-19 period. In this review, we aim to discuss and expand some clinical points related to GC excess that may have an impact on COVID-19 infection, in terms of both contagion risk and clinical outcome. This document is addressed to all specialists who approach patients with endogenous or exogenous GC excess and COVID-19 infection.

## Introduction

Severe acute respiratory syndrome due to coronavirus SARS-CoV2, or COVID-19, has recently been identified to be a cause of severe pneumonia, with potential evolution to acute respiratory distress syndrome (ARDS), further complicated by cardiovascular and renal injury, particularly in older patients with metabolic comorbidities, such as obesity, hypertension and diabetes, in which higher morbidity and mortality have been observed [1, 2]. Metabolic alterations are common clinical features of Cushing’s syndrome (CS), a complex and challenging disease characterized by chronic glucocorticoid (GC) excess [3–5]. CS can be exogenous, resulting from chronic administration of corticosteroids, or endogenous, due to adrenal overproduction of cortisol. In around 80% of cases, endogenous CS is caused by excessive adrenal stimulation from abnormally elevated ACTH levels, due to an ACTH-secreting pituitary tumour (Cushing’s Disease, CD) or by an extra-pituitary ACTH-secreting tumour (ectopic CS), whereas in around 20% of cases CS is related to autonomous, dysregulated cortisol secretion by the adrenal glands [3, 5, 6].

Aside from metabolic alterations, CS patients also experience increased susceptibility to infections, due to immune impairment [7]. Therefore, during a COVID-19 pandemic, it could be argued that CS patients may be at high risk of developing COVID-19 infection, as well as potentially experiencing a more severe clinical course due to their chronic metabolic comorbidities, which usually improve but are often not fully normalized after hypercortisolism resolution [6]. However, since CS has an estimated prevalence of around 40 cases per million and an estimated incidence of 0.7–2.4 cases per million per year, although the worldwide epidemiology has not been fully determined [8], up to now, there have been no reports of COVID-19 infections in CS patients.

The European Society of Endocrinology (ESE) has recently commissioned an urgent clinical guidance document on management of CS in a COVID-19 period [9]. In the current review, we aim to discuss and expand some clinical points related to GC excess that may have an impact on COVID-19 infection, in terms of both contagion risk and clinical outcome.

### Immune system dysregulation and infections

**Fig. 1:**
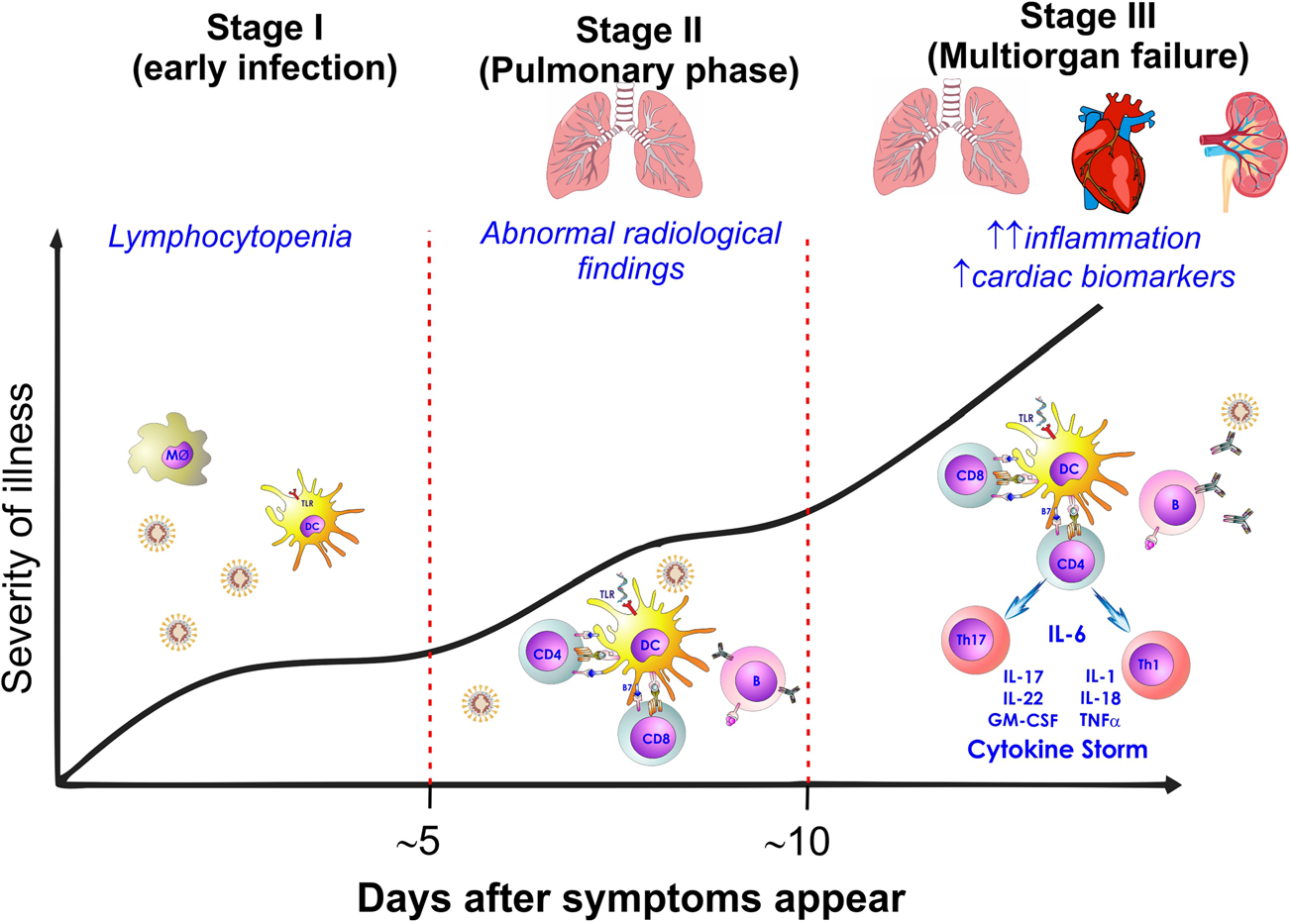
Role of glucocorticoid (GC) excess in immune system dysregulation and covid sars 2 infection. SARS-CoV2 achieves cell entry through an S high-affinity protein binding to the catalytic domain of the ACE 2 receptor that is highly expressed in cells in the respiratory tract. In innate immune response GC reduce natural killer (NK) cytotoxic action and classical activation of macrophages (M1) whereas intermediate and nonclassical monocyte levels, and macrophage alternatively activated (M2) characterized by a lower phagocytic activity are increased. GCs reduce antigen presentation and can also significantly influence the Th1/Th2 balance and induce apoptosis in mature T lymphocytes, producing a significant shift towards Th2 differentiation. Furthermore, GCs reduce the differentiation of Th17 cells favouring infection development. COVID-19 infection is associated with marked pro-inflammatory cytokine production and pro-inflammatory macrophage and granulocyte recruitment, resulting in a “cytokine storm” leading to a multiorgan failure. In addition adipose tissue is able to secrete pro-inflammatory cytokines, including IL-6 and TNF-α, that are therefore increased in obese patients. Similarly to CS patients in COVID-19 infection there is an increase in procoagulant factor levels including fibrinogen and D-dimers, mainly related to the excessive cytokine release, to the abnormal activation and recruitment of monocytes, macrophages, neutrophils and NET (Neutrophil Extracellular Traps) formation. Abbreviations: Baff: B cell activating factor; Baff-r: Baff receptor; CS: Cushing syndrome; DC: Dendritic cell; GCs: Glucocorticoids; GCR; glucocorticoid receptor; GM-CSF: Granulocyte-macrophage Colony-stimulating factor; H2O2: Hydrogen peroxide IL-: Interleukin-; MICA: MHC class I chain-related a; MPO: Myeloperoxidase; NO: Nitric oxide; TF: Tissue factor; Th: Thelper; TNF-α: Tumor necrosis factor-α

Chronic excessive GC exposure affects both innate and adaptive immune responses to infective states. Considering innate response during chronic GC excess, natural killer cytotoxic action on virally infected cells and complement classic and alternative activation pathways are reduced, whereas intermediate and nonclassical monocyte levels, characterized by lower phagocytic activity compared to classical monocytes, are increased [6, 7]. Moreover, neutrophil peripheral migration in damaged tissues, reactive oxygen species production and overall response to cytokines and inflammatory mediators, including lipopolysaccharides, are significantly impaired by prolonged GC exposure. Furthermore, an increase in pro-inflammatory cytokine secretion, including interleukine-6 (IL-6) and tumour necrosis factor-α (TNF-α), is observed in chronic GC excess, with persistent low-grade, inflammation-related tissue damage [7]. Therefore, GC may hamper the first-line response to external agents and consequent activation of the adaptive response. Indeed, a decrease is observed in the total number of T- and B-cells, mainly due to increased apoptosis in the early phases of lymphocyte development, as well as a reduction in T-helper 1 cell activation by dendritic cells, favouring opportunistic and intracellular infection development [7]. On the basis of these evidences, GC excess-related immune alterations may lead to an increased infective risk for CS patients, in terms of infective susceptibility and prognosis. Indeed, CS patients reportedly experience a more severe and protracted clinical course of respiratory viral infections, including influenza or adenovirus-related infections, and may be more prone to developing various common fungal, bacterial and viral infections, including herpes simplex, herpes zoster, cytomegalovirus and Epstein-Barr virus [6, 10, 11]. Moreover, it should be noted that the development of ARDS during COVID-19 infection is associated with marked pro-inflammatory cytokine production and pro-inflammatory macrophage and granulocyte recruitment, resulting in exaggerated cytokine release, notably Il-6 and TNF-α, also known as a “cytokine storm” leading to multi-organ failure [12] (Fig. 1). Unfortunately, patients experiencing increased cytokine levels usually have a poorer prognosis [13]. Due to the pro-inflammatory state of CS patients, it could be speculated that these patients may be more prone to progression from pneumonia to ARDS, since inflammatory cytokine levels are already increased [7]. Nevertheless, in CS patients the rise in cytokine levels associated with exposure to external agents is significantly hampered, probably because of persistently elevated pro-inflammatory cytokine secretion [6]. Therefore, it cannot be excluded that CS patients may also be less likely to develop COVID-19 infection complications related to the cytokine storm, including ARDS. COVID-19 infection can also lead to dysregulation in the levels of lymphocyte subsets [14]. Lymphopenia is very common in patients with COVID-19 infection, with a decrease in CD4+ T-lymphocytes, CD8+ T-lymphocytes, B-lymphocytes, and natural killer levels [14]. Lymphopenia is also reported in CS patients, in whom both T cell and B cell maturation is impaired. In particular, CS patients experience an unbalance in T-helper cell differentiation, T-helper 1 cells, usually involved in viral response, being less likely to develop compared to T-helper 2 cells [7]. Since a significant decrease in CD8+ T cells compared to other lymphocyte subsets during COVID-19 infection has been reported [14], it should not be excluded that the decrease in both CD4+ and CD8+ lymphocytes may worsen the COVID-19 infection prognosis in CS patients, in a synergistic way. Overall considered, it could be speculated that the chronic increase in pro-inflammatory cytokines observed in CS, together with impairment of lymphocyte maturation and differentiation, may lead to a worse COVID-19 infection prognosis in hypercortisolemic patients, as they may be more prone to developing excessive cytokine secretion and therefore progress from pneumonia to ARDS. At the same time, impairment of the cytokine rise during response to external agents in CS patients may also lead to a more stable clinical course, avoiding ARDS development. In clinical practice, GCs have been widely used during ARDS and are being used in patients with COVID-19 in addition to other drugs. At present, the current WHO guidance does not recommend routine GC treatment for COVID-19 pneumonia outside clinical trials [15]. However, some selected critically ill patients with COVID-19 would benefit from timely use of corticosteroids [16]. Finally, the use of GC in patients with COVID-19 pneumonia remains controversial [17, 18].

In conclusion, since no clear evidences are currently available, it could be reasonable to consider CS patients at high risk of contagion and to enhance preventive measures to reduce infective risk, including social distancing, the use of personal protection equipment, and frequent hand washing.

### Obesity

Up to 100% of patients with CS show weight excess, mainly associated with visceral obesity. Indeed, GC excess results in increased abdominal visceral adipose tissue deposition and reduced peripheral subcutaneous adipose depots, probably due to the different GC responsiveness of visceral fat compared to subcutaneous fat [6, 19–21]. Obesity represents an independent risk factor for development of severe COVID-19 infection, being associated with higher morbidity and mortality [2, 22]. The reason behind obese patients’ vulnerability to COVID-19 infection is still a matter of debate. It is known that adipose tissue is able to secrete pro-inflammatory cytokines, including Il-6 and TNF-α, which are therefore increased in obese patients. Moreover, in adipose tissue of obese mice an increased number of T-helper 1 and B-cells, actively promoting local pro-inflammatory states, has been observed [23]. Therefore, the pro-inflammatory state of obese patients may favour COVID-19 disease progression towards a more severe prognosis, which may be further worsened in CS patients, where a GC excess-related pro-inflammatory state is already present. At the same time, it should be noted that SARS-CoV2 achieves cell entry through an S (spike) high-affinity protein binding to the catalytic domain of the angiotensin-converting enzyme (ACE) 2 receptor [24], which is highly expressed in adipose tissue [25]. Consequently, it could be speculated that adipose tissue may serve as a reservoir for SARS-CoV2, as has been observed for other infective agents, including adenovirus, influenza A virus, human immunodeficiency virus, cytomegalovirus, Trypanosoma gondii and *Mycobacterium tuberculosis* [26]. As adipose tissue is difficult for antiviral drugs to reach, it cannot be excluded that the constant release of viral replicas from the adipose tissue reservoir may interfere with COVID-19 infection treatment, delaying its resolution and favouring a worse prognosis. Based on these evidences, CS patients with visceral obesity should be carefully monitored in case of COVID-19 infections, due to the increased morbidity and mortality in obese patients, and a more aggressive therapy should be considered, since the adipose tissue reservoir may lead to decreased therapeutic efficacy.

### Diabetes mellitus

About 11–47% of patients with CS develop diabetes mellitus, mainly due to decreased insulin sensitivity and impairment of beta-cell function induced by GC excess [6, 27–29]. Diabetes mellitus is associated with poorer outcomes in COVID-19 infection, together with a higher prevalence of lymphocytopenia and higher expression of pro-inflammatory cytokines, including IL-6 and TNF-α, compared to non-diabetic patients [30–33]. Such evidences may be related to the increased levels of inflammatory biomarkers in diabetic patients, mainly associated with the previously mentioned abnormal cytokine secretion by excessive adipose tissue which is commonly present in diabetic patients [34]. Moreover, diabetic patients usually experience impaired neutrophil and macrophage activity, as well as decreased T cell lymphocyte function, further increasing the risk for a more severe disease course [35]. Therefore, it would be reasonable to consider CS patients with diabetes to have an increased risk of severe COVID-19 infections, because of the potential progression to ARDS induced by excessive pro-inflammatory cytokines and by innate and adaptive immune system impairment. In addition, COVID-19 diabetic patients have shown higher levels of D-dimer, a coagulation activity marker, compared to non-diabetic patients, suggesting a greater tendency to hypercoagulability [33]. Since CS patients already experience a higher thromboembolic risk compared to healthy subjects [6, 36] and disseminated intravascular coagulation (DIC) may represent a life-threatening complication of COVID-19 infection, strict monitoring of coagulation parameters and early thromboembolic prophylaxis should be started in diabetic CS patients in case of COVID-19 infection. Interestingly, some studies tend to suggest that COVID-19 infection may worsen pre-existing diabetes or predispose non-diabetic subjects to diabetes [30]. The main mechanism favouring the close relationship between diabetes mellitus and COVID-19 is the wide expression of ACE-2 receptors in endocrine pancreas, inducing insulin resistance and impaired insulin secretion [37]. In patients with SARS, whose clinical course resembles that of the COVID-19 infection, an increase in fasting blood glucose levels was demonstrated [38]. In addition, the expression of ACE2 protein is strong in pancreatic islets, meaning that SARS-CoV2 might cause direct damage to islets, resulting in diabetes [39]. A vicious circle of diabetes and COVID-19 may contribute to a severe prognosis [39].

Special attention should be paid to antidiabetic treatment for all diabetic patients with COVID-19 infection. All agents potentially impacting on dehydration such as metformin, SGLT-2 inhibitors and GLP-1 agonists (due to gastrointestinal adverse events) should be suspended and renal function should be closely monitored for these patients. Treatment with sulphonylureas should be avoided due to the high risk of hypoglycaemic events, while insulin therapy should be encouraged to reach adequate and rapid glycaemic control [40]. Based on the evidence that the DPP4 is the functional receptor for the virus responsible for Middle East Respiratory Syndrome (MERS), a potential role of DPP4 in patients with diabetes mellitus and COVID-19 infection has been hypothesized, suggesting a better course of the COVID-19 infection in patients with diabetes mellitus treated with DPP4 inhibitors [41]. However, currently there are no reports showing this favourable association in patients with COVID-19.

### Arterial hypertension

Systemic arterial hypertension is a very common clinical feature of CS, occurring in up to 93% of patients [6, 36, 42–44]. GC excess causes hypertension by several mechanisms such as an increase in mineralocorticoid activity, activation of the renin-angiotensin system, increase in vascular reactivity to vasoconstrictors, enhancement of β-adrenergic receptor sensitivity to catecholamines and suppression of the vasodilatory system [36, 43, 44]. Hypertension has been associated with a more severe COVID-19 clinical course, although it is unclear whether being hypertensive or having uncontrolled hypertension represents the main risk factor [45]. Up to now, no clear evidences have explained the increased severity of COVID-19 infections in hypertensive patients. A huge, worldwide debate has arisen in the last few months regarding the potential impact of ACE inhibitors or angiotensin receptor blockers (ARB) on COVID-19 infection. Indeed, it has been observed that ACE inhibitors or ARBs treatment may increase ACE2 expression [46], thus potentially increasing infective risk for COVID-19 infection. However, none of three recent studies showed evidence of harm with continued use of ACE inhibitors and ARBs [47–50]. Moreover, it has been demonstrated that use of ACE inhibitors, by reducing formation of angiotensin II, or use of ARBs, by antagonizing the action of angiotensin II by blocking angiotensin AT1 receptors, could actually contribute to reducing systemic inflammation, particularly in the lungs, heart and kidneys [51]. Furthermore, increased soluble ACE2 blood stream expression could bind SARS-CoV2, preventing tissue damage to ACE2-bearing organs, although with the risk of a more prolonged disease due to slower virus elimination [52]. A recent cohort study showed that the use of ACE inhibitors or ARBs was associated with significantly improved survival in patients with hypertension hospitalized with COVID-19 [53]. In conclusion, the European Society of Hypertension (ESH) COVID-19 Task Force affirms that the available evidence does not support a deleterious effect of RAS blockers in COVID-19 infections [54].

On the basis of these evidences, hypertensive CS patients should nevertheless be considered at high risk for severe COVID-19. The recent ESE guidance states that there is no reason to discontinue ARBs in stable hypertensive CS patients facing COVID-19 but is against starting these drugs until their influence on susceptibility to SARS-CoV2 infection has been clarified [9]. However, currently there are no studies that address the potential benefits and harms of initiating ACE inhibitors or ARBs as treatment for patients with COVID-19.

### Hypercoagulability

CS is associated with a high risk of thromboembolic events. Many alterations of coagulation and fibrinolysis can occur in CS, including an increase in factor VIII, fibrinogen, and von Willebrand factor levels, and a shortening of the activated partial thromboplastin time. In addition, patients with CS generally show high platelet levels [6, 36, 55]. Similarly, patients with COVID-19 infection show coagulation abnormalities, characterized by increases in procoagulant factor levels including fibrinogen and D-dimers, which are correlated with high mortality [56]. These evidences seem to be related to excessive cytokine release, mainly due to abnormal activation and recruitment of monocytes, macrophages and neutrophils. Indeed, the innate system cells may increase thromboembolic risk both through direct thrombin activation and indirect activation of the exogenous coagulation pathway by local tissue factor secretion [56, 57]. Therefore, a hypercoagulable state is observed in COVID-19 patients, leading in the worst cases to life-threatening disseminated intravascular coagulation, which is associated with worse prognosis and a higher risk of death [2, 56, 57]. On the basis of these evidences, it would be reasonable to start proper thromboembolic prophylaxis in CS patients during COVID-19 infection, especially in the case of reduced mobility due to hospitalization, to prevent potential worsening of the infective prognosis [58]. Among antithrombotic drugs, low molecular weight heparin (LMWH) should be considered the treatment of choice, as recent evidences suggest a more favourable prognosis and clinical course in patients treated with LMWH during COVID-19 infections [59].

### Myopathy

Cushing’s myopathy has not been associated with respiratory failure as a presenting symptom. However, CS usually causes profound atrophy of skeletal muscle without inducing tissue necrosis, with a higher impact on proximal muscle [3, 60]. Interestingly, a case of respiratory failure due to CS myopathy has been described [61], which was resolved when treatment of hypercortisolism was started [62, 63]. Although rarely, CS can be associated with respiratory insufficiency, and therefore it should be considered as a potential confounding factor for a suspicion of COVID-19 infection in this subset of patients.

### Depression and neuropsychiatric disorders

COVID-19 appears to significantly burden the mental health of all patients and might cause delirium in a significant proportion of patients in the acute stage [64, 65].

Neuropsychiatric disorders are severe complications of CS. GC excess causes behavioural disorders which are initially represented by insomnia and euphoria and later by depression. The most important and life-threatening psychiatric complication in CS patients is major depression (50–80% of cases), but other psychiatric disorders (anxiety disorders, mania, and hypomanic episodes) are also frequent. It is important to note that in CS patients suicide, alongside cardiovascular and cerebrovascular disease, thromboembolism, infectious diseases or sepsis, represent a main cause of death [3, 6]. Besides normalization of cortisol levels, cognitive behavioural therapies and antidepressant drugs can be useful in treating CS-associated psychiatric disorders. Finally, in cases of severe anxiety, benzodiazepines can be used.

We think this is an important point which clinicians should take into consideration both in the acute phase of the disease and in the recovery for possible long-term neuropsychiatric consequences (depression, anxiety, fatigue, post-traumatic stress disorder).

## Therapeutic approach to patients with active Cushing’s syndrome during a COVID-19 pandemic

### Surgery

Surgery is the first-line treatment in CS, regardless of its aetiology [66, 67], but it should be considered with caution during a COVID-19 pandemic. Surgery procedures require hospitalization, which may be associated with an increased risk of contagion, considering that hospital-related COVID-19 transmission was suspected in 41% of patients and that 12.3% of infected patients were already hospitalized [68]. Moreover, aside from CS-related immune impairment [7], surgical procedures may lead to immune system alterations per se, due to excessive systemic inflammatory response related to surgically induced tissue damage and to anaesthetics and opioid inhibition of natural killers, neutrophils, and lymphocyte activity and survival [69]. Therefore, an increased risk for COVID-19 infection should be considered in CS patients being hospitalized or undergoing surgery, in particular in malignant CS patients, where cancer-related immune suppression may further enhance infective susceptibility [70]. However, according to the ESE clinical guidance any patient with a malignant CS needs an individualized evaluation of the risks vs. benefits of cancer therapy during COVID-19, either with surgery and/or medical approaches [9]. Additionally, it should be noted that all patients who have undergone major surgery have an increased risk of thromboembolic events [71], as do CS patients [6, 36], associated with a worse prognosis during COVID-19 infections [2], thus representing a further reason to defer surgical treatment of CS patients to the end of a COVID-19 pandemic. In ACTH-dependent CS, when other treatment modalities are not suitable, available or effective [66, 67] bilateral adrenalectomy provides an immediate resolution of hypercortisolism, but it also leads to iatrogenic, chronic adrenal insufficiency, which is associated with higher morbidity and mortality compared to healthy subjects, due to the high risk of infections [72–74]. Therefore, also considering the surgery-related increased infective and thromboembolic risk, bilateral adrenalectomy should be avoided during a COVID-19 pandemic, preferring medical treatment for emergency management of very severe CS.

### Radiotherapy

Radiotherapy is a second-line or third-line treatment in CS patients not suitable for or refusing surgery and in recurrent patients not responsive to medical treatment. It is especially useful in CD patients for hormonal control and in a malignant CS adjuvant and palliative setting [66, 67, 75–77]. In patients with malignant CS, the radiotherapy approach should be evaluated in the oncologic setting, also taking into account the required multiple hospital admission [75, 76], which may lead to a higher contagion risk, whereas hypercortisolism control should be preferably reached through alternative treatment modalities.

### Medical therapy

Medical therapy is a second-line or third-line treatment for CS patients not suitable for or refusing surgery, in recurrent CS patients and in CD patients waiting for radiotherapy efficacy, as bridge treatment [66, 67]. Medical therapy is represented by adrenal steroidogenesis inhibitors, including ketoconazole, metyrapone, osilodrostat, mitotane, and etomidate, directly affecting adrenal cortisol production. The glucocorticoid receptor (GR) antagonist mifepristone impairs cortisol binding to GR and mainly acts on clinical comorbidities; pituitary-directed agents, including pasireotide and cabergoline, target pituitary source of ACTH excess. Adrenal steroidogenesis inhibitors and mifepristone may be useful in all CS aetiology, whereas pituitary-directed agents should only be considered in CD and in some cases of ectopic ACTH [67]. In malignant CS chemotherapy could possibly be considered as a medical therapy for CS, as the reduction in the tumoral masses is usually associated with a reduction in hormonal hypersecretion, but their analysis goes beyond the aim of the current paper, and their use should be carefully evaluated by the referral oncologists.

During a COVID-19 pandemic, medical therapy may represent an interesting and effective approach in CS patients, since it may be able to promptly reduce circulating cortisol levels and therefore to improve clinical comorbidities with a potential impact on the COVID-19 infection course. However, specific evaluation regarding the pharmacodynamics and safety profile of every drug should be performed. Among adrenal steroidogenesis inhibitors, ketoconazole, metyrapone, and recently available osilodrostat should be considered as the drugs of choice in CS during a COVID-19 pandemic, due to their high efficacy, hormonal control being obtained in many treated patients, and to their rapid action onset, usually requiring just a few days to normalize cortisol levels [67, 77]. However, metyrapone treatment may be associated with hypokalemia and worsening of hypertension [67, 78], which can negatively impact the course of a COVID-19 infection [2]. Thus close blood pressure monitoring in treated CS patients is always required. At the time of monitoring metyrapone effects, it should be kept in mind that cortisol levels should be measured with chromatography to avoid overestimation due to assay cross-reactivity with the precursor 11-deoxicortisol. Commercially available assays can underestimate the presence of adrenal insufficiency, which can be even more perilous than CS in the case of infection. Nevertheless, metyrapone is probably the best choice to start treatment of hypercortisolism during COVID-19. Because of accumulation of cortisol precursors, a mass spectrometry assay is needed to measure cortisol during treatment with metyrapone. At the same time, it should be noted that COVID-19 infection has been associated with liver injury, mainly represented by a transient increase in serum amminotranspherase [79], which may be present in up to 15% of CS patients treated with ketoconazole [67, 78]. Therefore, metyrapone can be preferred over ketoconazole in CS patients with biochemical evidences of liver injury during COVID-19 infection. In addition, drug-drug interactions are frequent during ketoconazole treatment and should be taken into account in patients with active infection who require other treatments. According to ECE clinical guidance, liver function should be monitored every month (deviating from EMA guidance for weekly testing) for the start of therapy or following a dose increase [9]. In addition, ketoconazole interacts with many drugs by interfering with CYP3A4 and may increase the risk of QT prolongation with fatal events. Osilodrostat is a promising novel drug, recently approved for treatment of CS. It is an orally potent inhibitor of CYP11B2, which also inhibits CYP11B1 at higher doses [80].

Analysing the remaining steroidogenesis inhibitors, mitotane should not be preferred, due to its slow action onset and the potential occurrence of permanent adrenal insufficiency and leukopenia, which may favour COVID-19 contagion [67, 78], whereas etomidate could be a valuable option in the emergency setting, as it may lead to rapid and effective control of hypercortisolism [67, 81], although hospitalization is required to avoid treatment-induced adrenal insufficiency, potentially increasing infective risk, and only a few cases have been treated until now. The glucocorticoid receptor antagonist mifepristone may be considered a potential option for CS treatment during a COVID-19 pandemic, since an improvement in CS metabolic comorbidities has been observed in up to 75% of patients [67, 82]. However, occurrence or worsening of hypertension, a risk factor for COVID-19 infection [2], was observed in about 25% of treated patients because of the paradoxical activation of the mineralocorticoid receptor by excessive circulating cortisol levels [67, 82]. Due to the non-univocal effects on hypertension, mifepristone should be considered as a secondary option for CS treatment during a COVID-19 pandemic, requiring careful blood pressure level monitoring. In CD patients, the pituitary-directed agents pasireotide and cabergoline may also be evaluated as treatment options for hypercortisolism control during a COVID-19 pandemic, although their intermediate efficacy, with hormonal control reached in 25% and 31% of treated patients, respectively [67], should limit their use as second-line approaches except for patients with mild CS [28, 83]. In particular, pasireotide should not be preferred, due to hyperglycaemia-related adverse events, which may occur in up to 73% of treated patients [67, 84–86], and to the higher morbidity and mortality in COVID-19 patients with diabetes mellitus [2].

If patients are already on cortisol-lowering medications, there is no need to shift from one treatment to another, except in the case of hormonally active disease.

CS patients in medical treatment to control hypercortisolism should be monitored closely for signs and symptoms of adrenal insufficiency. Finally, according to ECE clinical guidance, we recommend giving a stress dose of glucocorticoid in a controlled CS patient on medical treatment in the case of SARS-Cov2 infection [9].

## Exogenous Cushing’s syndrome due to glucocorticoid administration in the COVID-19 pandemic

The correlation between CS and rheumatic disease is complex: on the one hand the presence of primitive hypercortisolism could mask expression of a rheumatic disease [87]; on the other hand, exogenous administration of GCs in rheumatic diseases could induce a iatrogenic form of CS. The scientific rationale for the use of GCs in inflammatory and/or autoimmune diseases is their anti-inflammatory and immunosuppressive effect. This effect is expressed through inhibitory modulation of the innate and adaptive immune system. For these reasons, steroids have been used in the advanced stages of COVID-19 infection with characteristics of respiratory distress syndrome in relation to the potential anti-inflammatory and immunomodulatory effects. In this context, the chronic use of steroids in rheumatic diseases during a COVID-19 pandemic is particularly important. Furthermore, adverse prognostic factors associated with COVID-19 infection (age > 65 years, hypertension, diabetes mellitus, chronic kidney disease), are frequently present in patients suffering from rheumatic diseases, above all those continuously treated with steroid therapy [88–91]. GCs play a pivotal role in management of many inflammatory rheumatic diseases such as systemic autoimmune diseases, inflammatory arthritis, polymyalgia rheumatica and vasculitis. Higher initial dosages are often used in patients with vasculitis and inflammatory myopathies. The duration of steroid therapy changes according to the type of rheumatic disease. However, recently GC doses have been gradually decreased in relation to the frequent use of steroid-sparing immunosuppressive drugs [92]. In rheumatoid arthritis, GCs represent pivotal drugs in the management of arthritis flares [92, 93], though they are used at a lower dosage and for shorter periods of time. However, despite the introduction of next-generation steroid-sparing drugs, some patients continue to use GCs and may develop iatrogenic CS [92]. Most patients with polymyalgia rheumatica (PMR) or giant cell arteritis (GCA) are treated with medium-to-high doses of prednisolone (up to 15 mg for PMR and 60 mg for GCS) for 6 months to several years [94]. A recent study has shown an increase in infectious risk in patients with PMR treated with medium-low doses of GCs [95]. In this group of patients, the use of GCs could be responsible for iatrogenic CS and delay the incubation period of COVID-19 infection. Management of gout flares with presently available agents can be more challenging due to potential nephrotoxicity and/or contraindications. In such cases the use of GCs could represent a risk of infection in the setting of other common comorbid conditions (hypertension, diabetes) [96]. GCs are anchor drugs in inflammatory myopathies. Higher initial dosages are often used (mostly 0.5–1 mg/Kg/die of prednisone) for these diseases [97]. Furthermore, in the case of severe pulmonary involvement, pulse therapy is applied [97]. Dermatomyositis (DM) is one of the causes of macrophage activation syndrome (MAS). Patients with MAS exhibit various symptoms, such as spiking fever, splenohepatomegaly, cytopenia, coagulopathy, respiratory distress syndrome and hyperferritinemia, caused by abnormal activation of the immune system and overproduction of pro-inflammatory cytokines, leading to excessive activation of macrophages [98]. These patients may present a respiratory distress pattern similar to severe manifestations of COVID-19 [99]. In such cases the use of GCs and biological drugs (anti-IL6, anti-IL1, JAK inhibitors) could be effective. GCs are often used in vasculitis with severe organ involvement [100]. Patients with kidney and/or pulmonary involvement (i.e. ANCA-associated vasculitis) and with concomitant use of immunosuppressive drugs could present more rapid clinical progression of COVID-19 infection, similarly to transplant patients [100, 101]. There are no studies to date showing a higher incidence of rheumatological diseases in patients with COVID-19 infection. Moreover, the few recorded cases treated with low doses of prednisone (<5 mg/day) do not show clinical deterioration [102, 103]. For these reasons, the indications regarding GCs in rheumatological diseases, with the exception of respiratory distress conditions, suggest use of low-dose GCs in order to prevent a disease flare without significantly increasing the infectious risk [104].

The recent published American College of Rheumatologic COVID-19 clinical guidance affirms that standard glucocorticoid administration should be continued, while abrupt discontinuation of treatment must be avoided, given the high risk of HPA axis suppression [104]. The task force also endorsed the use of low-dose glucocorticoids when clinically indicated [104].

## Glucocorticoid treatment in COVID-19 infection

Current recommendations on COVID-19 infection treatment are based on the use of oral or intravenous dexamethasone. The benefit of steroid treatment has only been demonstrated in patients requiring oxygen therapy with or without mechanical ventilation. Indeed, in the randomized RECOVERY trial dexamethasone reduced mortality by one-fifth in patients requiring non-invasive oxygen therapy, and by one-third in those requiring mechanical ventilation. Dexamethasone also reduced hospital stay length and progression to needing invasive mechanical ventilation [105]. A recent metanalysis from the WHO Rapid Evidence Appraisal for COVID-19 Therapies (REACT) Working Group pooled data from 7 trials (RECOVERY, REMAP-CAP, CoDEX, CAPE COVID, and 3 additional trials) totalling 1703 patients showing that the 28-day mortality was lower in patients randomized to corticosteroids. The association between administration of corticosteroids and reduced mortality was similar for dexamethasone and hydrocortisone, suggesting the benefit was a general class effect of glucocorticoids and not specific to any particular corticosteroid, it was similar with lower- vs. higher-dose corticosteroid regimens and among patients with fewer vs. greater than 7 days of symptoms at randomization [106].

Therefore, administration of steroids is clearly associated with benefit among critically ill patients with COVID-19, although the exact threshold at which an individual patient should be prescribed corticosteroids remains unclear. However, although it might be thought that patients with endogenous or exogenous hypercortisolism may be protected, actually it is not the case. Indeed, an acute effect of corticosteroids has anti-inflammatory properties and mild side effects with a benefit on COVID-19 infection course, while chronic and prolonged hypercortisolism can result in serious adverse events with metabolic and cardiovascular complications and HPA axis suppression, which can play a unfavourable role in the course of COVID-19 infection.

## Conclusions

**Fig. 2:**
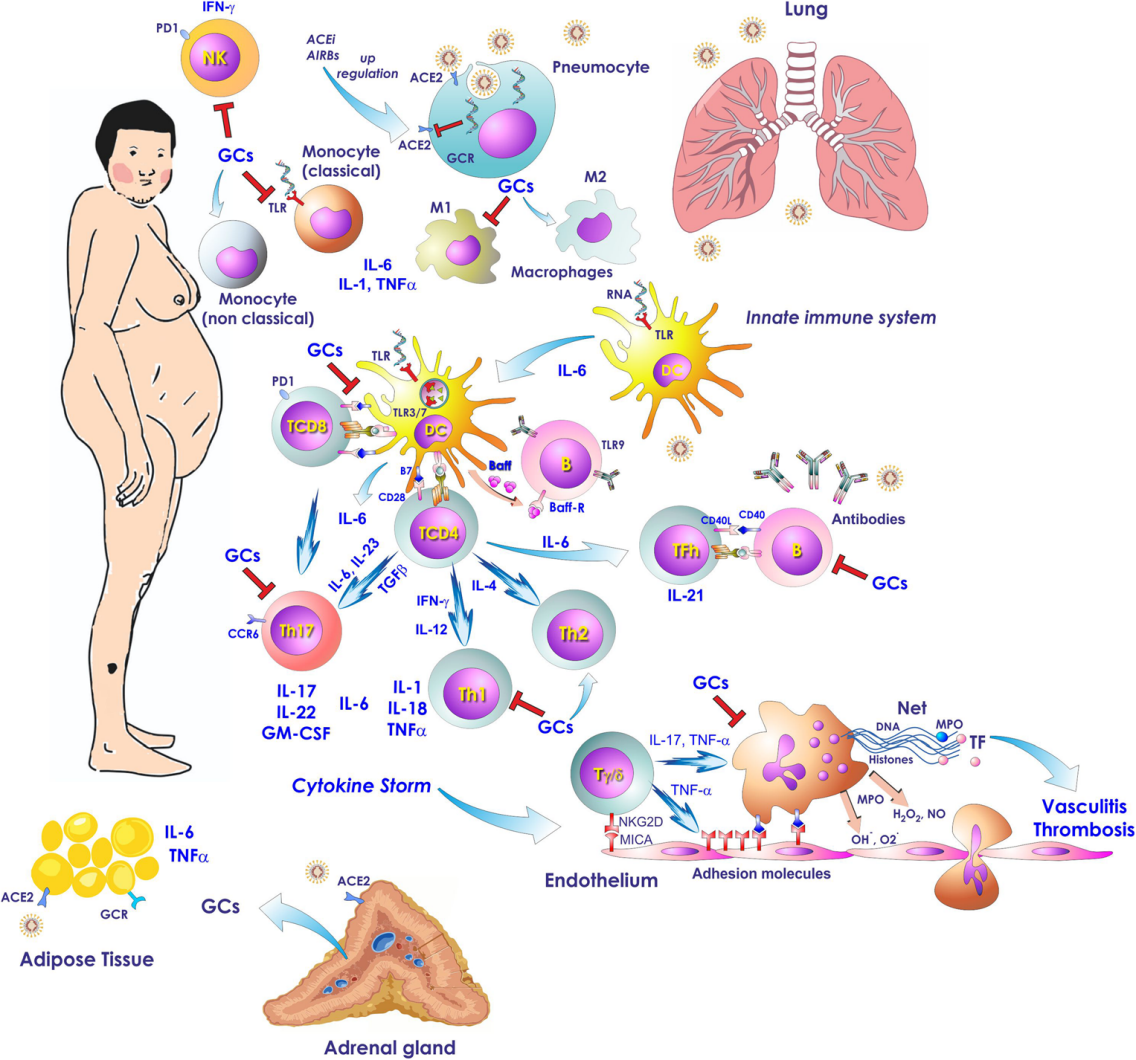
Hypothetical stages of evolution of Covid-19 infection. The initial phase is characterized by the entry of the virus, through the respiratory tract. There is an increase of CRP and CBC may reveal lymphopenia and neutrophilia. In the second stage there is viral multiplication and localized pulmonary infection with abnormal radiological findings. Blood test reveal lymphopenia and elevated transaminases. In a minority of patients there is a transition to a third phase characterized by systemic inflammation induced by the cytokine storm with a respiratory distress pattern, cytopenia, coagulopathy and multiorgan failure. Abbreviations: CBC: Complete blood count; CRP: C reactive protein; GM-CSF: Granulocyte-macrophage Colony-stimulating factor; IL- interleukin-; MØ: Macrophage; TNF-α: Tumor necrosis factor-α

Patients with CS have impaired immune response predisposing them to common viral infections, and probably also to COVID-19 infection. In addition to this higher susceptibility, CS patients can potentially develop a more severe course of infection due to the variety of metabolic and cardiovascular complications related to cortisol excess (Fig. 2). Till now no specific treatment for COVID-19 prevention is available. Preventive measures such as social distancing and hand washing will be our only weapon to tackle the virus spread among CS patients too. Close control of metabolic and cardiovascular complications associated with cortisol excess is of paramount importance to ameliorate patients’ prognosis during any phase of the disease.

Surgery, which is usually the first-tier option for all endogenous CS causes, should probably be deferred and patients managed with steroidogenesis inhibitors to reduce immunosuppression. This treatment should be started before the patient gets infected, as its utility during active and severe infection is questionable since it exposes the patient to the risk of adrenal insufficiency. Given the high risk of thrombotic events due to the disease itself, and the procoagulative push of COVID-19 infection, LMWH is strongly suggested in hospitalized CS patients. Metformin should be withdrawn to avoid lactic acidosis in all patients with severe infections and insulin therapy should be started for patients with uncontrolled diabetes mellitus. Standard GC administration should be continued in patients with rheumatological diseases on steroid therapy at as low a dose as possible, while abrupt discontinuation of treatment must be avoided, given the high risk of HPA axis suppression.
